# Impact of population based indoor residual spraying with and without mass drug administration with dihydroartemisinin-piperaquine on malaria prevalence in a high transmission setting: a quasi-experimental controlled before-and-after trial in northeastern Uganda

**DOI:** 10.1186/s12879-023-07991-w

**Published:** 2023-02-06

**Authors:** Dorothy C. Echodu, Adoke Yeka, Thomas Eganyu, Wycliff Odude, Fred Bukenya, Benjamin Amoah, Humphrey Wanzira, Kathryn Colborn, Richard C. Elliott, Suzanne E. Powell, Maxwell Kilama, Ronald Mulebeke, Joaniter Nankabirwa, Emanuele Giorgi, Mellisa Roskosky, Osborn Omoding, Samuel Gonahasa, Jimmy Opigo

**Affiliations:** 1 Pilgrim Africa, 8001 14th Avenue NE, Suite A, Seattle, WA 98115 USA; 2 Makerere University College of Health Sciences, School of Public Health, P.O. Box 7072, Kampala, Uganda; 3 Pilgrim Africa, Plot 8 Engwau Road, PO Box 577, Soroti, Uganda; 4 School of Public Health, Imperial College London, Sir Alexander Fleming Building, South Kensington Campus, London, SW7 2AZ UK; 5 University of Colorado Anschutz Medical Campus, 12631 East 17th Avenue, Aurora, CO 80045 USA; 6 Micron School of Materials Science and Engineering, Boise State University, Engineering Building, Suite 338, Boise, ID 83725 USA; 7 Infectious Diseases Research Collaboration, Kampala, Uganda; 8 Lancaster University Medical School, Centre for Health Informatics, Computing and Statistics, Lancaster, UK; 9 Johns Hopkins Bloomberg School of Public Health, Baltimore, MD USA; 10 National Malaria Control Division, Ministry of Health Uganda, Kampala, Uganda

**Keywords:** MDA, Malaria, IRS, High burden, Uganda, Controlled trial, Pirimiphos, Dihydroartemisinin

## Abstract

**Background:**

Declines in malaria burden in Uganda have slowed. Modelling predicts that indoor residual spraying (IRS) and mass drug administration (MDA), when co-timed, have synergistic impact. This study investigated additional protective impact of population-based MDA on malaria prevalence, if any, when added to IRS, as compared with IRS alone and with standard of care (SOC).

**Methods:**

The 32-month quasi-experimental controlled before-and-after trial enrolled an open cohort of residents (46,765 individuals, 1st enumeration and 52,133, 4th enumeration) of Katakwi District in northeastern Uganda. Consented participants were assigned to three arms based on residential subcounty at study start: MDA+IRS, IRS, SOC. IRS with pirimiphos methyl and MDA with dihydroartemisinin- piperaquine were delivered in 4 co-timed campaign-style rounds 8 months apart. The primary endpoint was population prevalence of malaria, estimated by 6 cross-sectional surveys, starting at baseline and preceding each subsequent round.

**Results:**

Comparing malaria prevalence in MDA+IRS and IRS only arms over all 6 surveys (intention-to-treat analysis), roughly every 6 months post-interventions, a geostatistical model found a significant additional 15.5% (95% confidence interval (CI): [13.7%, 17.5%], Z = 9.6, p = 5e−20) decrease in the adjusted odds ratio (aOR) due to MDA for all ages, a 13.3% reduction in under 5’s (95% CI: [10.5%, 16.8%], Z = 4.02, p = 5e−5), and a 10.1% reduction in children 5–15 (95% CI: [8.5%, 11.8%], Z = 4.7, p = 2e−5). All ages residents of the MDA + IRS arm enjoyed an overall 80.1% reduction (95% CI: [80.0%, 83.0%], p = 0.0001) in odds of qPCR confirmed malaria compared with SOC residents. Secondary difference-in-difference analyses comparing surveys at different timepoints to baseline showed aOR (MDA + IRS vs IRS) of qPCR positivity between 0.28 and 0.66 (p < 0.001). Of three serious adverse events, one (nonfatal) was considered related to study medications. Limitations include the initial non-random assignment of study arms, the single large cluster per arm, and the lack of an MDA-only arm, considered to violate equipoise.

**Conclusions:**

Despite being assessed at long time points 5–7 months post-round, MDA plus IRS provided significant additional protection from malaria infection over IRS alone. Randomized trials of MDA in large areas undergoing IRS recommended as well as cohort studies of impact on incidence.

*Trial registration*: This trial was retrospectively registered 11/07/2018 with the Pan African Clinical Trials Registry (PACTR201807166695568).

**Supplementary Information:**

The online version contains supplementary material available at 10.1186/s12879-023-07991-w.

## Background

Globally, progress against malaria morbidity has stalled, with the remaining burden concentrated in eleven countries, including Uganda [1]. Uganda is a stably endemic, high burden country with, as of 2020, the third highest number of malaria cases in the world [2, 3]. After some years of low movement against key malaria metrics [4], the country made great strides in the decade between 2009 and 2019: doubling insecticide treated net (ITN) ownership and use, maintaining a commitment to indoor residual spraying (IRS) in high transmission areas [5–8], emphasizing early diagnosis and prompt treatment of uncomplicated malaria with artemisinin combination therapies (ACTs), intermittent preventive treatment for pregnant women (IPTp) [9, 10], and improving national surveillance [11]. These efforts, along with growing urbanization and greater access to improved housing [12–15], resulted in dramatic national reduction in microscopy prevalence of children under 5 from 42% in 2009 [16] to 9% in 2018 [17]. In recent years, however, this progress is slowing. Further gains must come from accurately targeting control combinations towards remaining pockets of high transmission, where most national cases occur [18, 19].

While vector control remains the primary way to reduce malaria burden and transmission, chemoprevention may also have a role in high burden regions where progress has faltered [20, 21]. A growing enthusiasm to use chemoprevention in control settings is emerging. It is supported by evidence from, for example, China, who for nearly 40 years (1960–1999) used both radical treatment and mass drug administration (MDA) of antimalarials as primary malaria interventions. MDA was specifically targeted to reduce disease burden, with annual doses administered tracking the level of burden [22]. During this time, national malaria transmission fell from hyperendemic to pre-elimination levels, and malaria cases shrank from an estimated 30 million per annum in the 1940 s to a little over 30,000 in 2000 [23]. In May of 2021, the WHO granted China official status as a malaria free country [24, 25]. More recently, there has been a general call for new evidence on MDA in high burden settings, as a tool with untapped potential [1].

MDA is a full course of an antimalarial treatment given to the whole population in a given area at approximately the same time, irrespective of symptoms or infection status and with the exception of individuals for whom the medicine is contraindicated [26, 27]. In order for MDA to be effective, high coverage is essential [28, 29] and therefore good engagement with stakeholders is required. For maximum impact, MDA should be performed with an artemisinin-combination therapy (ACT) [30]. Dihydroartemisinin-piperaquine (DP) is often preferred for its efficacy, safety profile, and long post-treatment prophylactic protection [31, 32]. In 2022, the WHO announced new guidelines on malaria including a conditional recommendation for use of MDA in high transmission contexts for burden reduction, noting it “should be targeted at moderate to high transmission settings, regardless of seasonality [33]”. The guidelines encourage the use of a combination medicine other than the one used for frontline control, and note that the impact of MDA on clinical malaria will be highest and most cost effective in high transmission contexts, reducing as burden level decreases. Evidence considered for the decision showed impact on clinical malaria 1–3 months after MDA, with moderate certainty, but evidence for any impact of MDA on parasite prevalence in the 12 months following MDA was mixed, and of low (no impact) or very low (potential impact) certainty [34].

Absent of concurrent vector control, the duration of MDA impact on parasite prevalence in a high transmission setting is expected to be limited; rebound is predicted to occur rapidly after a transient period of chemoprophylactic prevention [35, 36]. A clinical trial of intermittent preventive treatment in schoolchildren (IPTsc), conducted in a high transmission region of Uganda, underlined this effect: chemoprevention was extremely protective when ACTs were administered at monthly intervals, reducing incidence and prevalence in intervention subjects relative to placebo by 96% and 94% respectively, but was much less effective in suppressing clinical incidence if given quarterly [37]. Similarly, in a large cluster-randomized trial of MDA in Zambia, with asynchronous vector control present in the study region, the effects of an MDA campaign after two rounds was estimated to last for 3–4 months [38]. In this case, MDA was shown to reduce cumulative incidence relative to control in a cohort [39, 40] in a high transmission setting; however, the differential impact of the MDA on prevalence was not significant in high burden areas at survey points 3–4 months after the campaign [40].

The synchronous application of MDA and vector control could increase the impact of MDA on prevalence. Prior work noted a robust impact of combining MDA with vector control, showing the strategy effective for burden reduction in high transmission regions, though not for achieving complete elimination [41]. Recent modeling has predicted complementary impact of MDA when added to IRS, especially if applied simultaneously, with the IRS protecting and effectively extending the duration of the MDA’s prophylactic effect for 6 months or more [35]. In Haiti, a low to moderate transmission quasiexperimental study compared the impact of targeted MDA + IRS on prevalence compared with LLINs and case management alone. The study showed that a single combined round (with intervention coverages of 33% and 51% for the IRS and MDA, respectively) showed a 68% greater relative reduction than the non-targeted areas when surveyed immediately after the round [42] Similarly, a cluster randomized trial in a low transmission setting in Namibia found that a combination of co-timed focal MDA and reactive focal vector control was much more effective than either of these interventions alone, and also found evidence for a positive synergistic effect when the interventions were co-deployed [43].

Because an MDA campaign initially removes so much infection in high transmission, the cooperative advantage is predicted to increase with transmission intensity, making the strategy of particular interest in high burden settings [36]. From a cost and compliance standpoint, the advantage of a single annual population-wide MDA as compared with monthly or seasonal chemoprophylaxis in children is compelling, but to date an experimental study has not investigated the impact of delivering IRS and MDA simultaneously in a high-burden context. Here, we evaluate the impacts of a co-timed MDA campaign with DP with IRS with pirimiphos methyl in a setting of high transmission and burden. Based on modeled predictions of the benefit of co-timing interventions, especially at long times after MDA implementation, the primary objective of this quasi-experimental study was to assess the impact on malaria prevalence of single rounds of population MDA+IRS at intervals five to seven months post-intervention, and to evaluate any additional protective impact of MDA when added to IRS over IRS alone and over standard of care (SOC). The comparison of MDA+IRS to IRS provides a picture of additional MDA impact, if any exists, at times well beyond the chemoprophylactic prevention period of the MDA campaign. This paper presents an overview of the interventions, procedures and primary prevalence outcomes.

## Methods

### Study design

This was a prospective controlled community intervention trial, quasi-experimental in the sense that residents were assigned to interventions based on sub-county of residence in an arbitrary but non-random fashion. The trial was conducted between October 2016 and June 2019, for a duration of 32 months, and had three assignment arms: MDA + IRS, IRS only, and SOC as the control arm, which included ITNs and case management at facilities with commodity support. SOC interventions were present in all three arms.

### Study setting

The study took place in Toroma County in the Katakwi District of northeastern Uganda, a high transmission region within the country deemed by the Uganda Ministry of Health (MOH) National Malaria Control Division (NMCD) to be suitable for targeted IRS. With a combined population of 46,765 in 2016, the project area consisted of three adjacent rural farming sub-counties bordering Lake Bisina. These three sub-counties served as the three arms of the study, with assignment taking place before study enrollment. Malaria transmission in this marshy area is dominated by *Anopheles gambiae sensu lato* (s.l.) and *Anopheles funestus s.l.* vector groups [44–46], and follows a bimodal rainfall pattern. The longer rainy season occurs between March–June, and a shorter season between October–November; malaria is hyperendemic year-round, with peak malaria transmission between May–July [47]. In the 5 years prior to the trial, all three sub-counties had very high numbers of annual reported malaria cases, roughly equivalent to their population, and took turns reporting the highest overall number of cases per capita in different years. All three sub-counties received free ITNs through national universal coverage campaigns conducted in November 2013 and again in April 2017 by Uganda MOH. Public health clinics in the study area follow the national malaria case management guidelines. None of the three sub-counties had received IRS in the 5 years prior to November 2016.

### Study interventions and arm assignment

This study had two main study interventions, IRS with pirimiphos methyl (Actellic 300 CS, Syngenta) and MDA with DP (Eurartesim, Sigma Tau). Population-wide IRS was conducted four times in two arms by the research team, spaced by roughly 8 months. In one of the arms, a single treatmemt dose of population-wide MDA was administered and co-timed with each IRS spray round.

IRS was conducted using Actellic 300 CS, a WHO recommended non-pyrethroid insecticide formulation of an existing organophosphate insecticide, pirimiphos-methyl, safe in use for humans and the environment and recommended by the WHO pesticide evaluations scheme (WHOPES) risk assessment [48]. Actellic has a longer residual effect than other non-pyrethroid IRS formulations (over 6 months), and was during the study period the primary insecticide used by MOH in partnership with Vectorlink. MDA employed DP (Eurartesim, Sigma Tau), a pre-qualified malaria treatment medicinal product according to the United Nations pre-qualification program managed by the WHO.

**Fig. 1:**
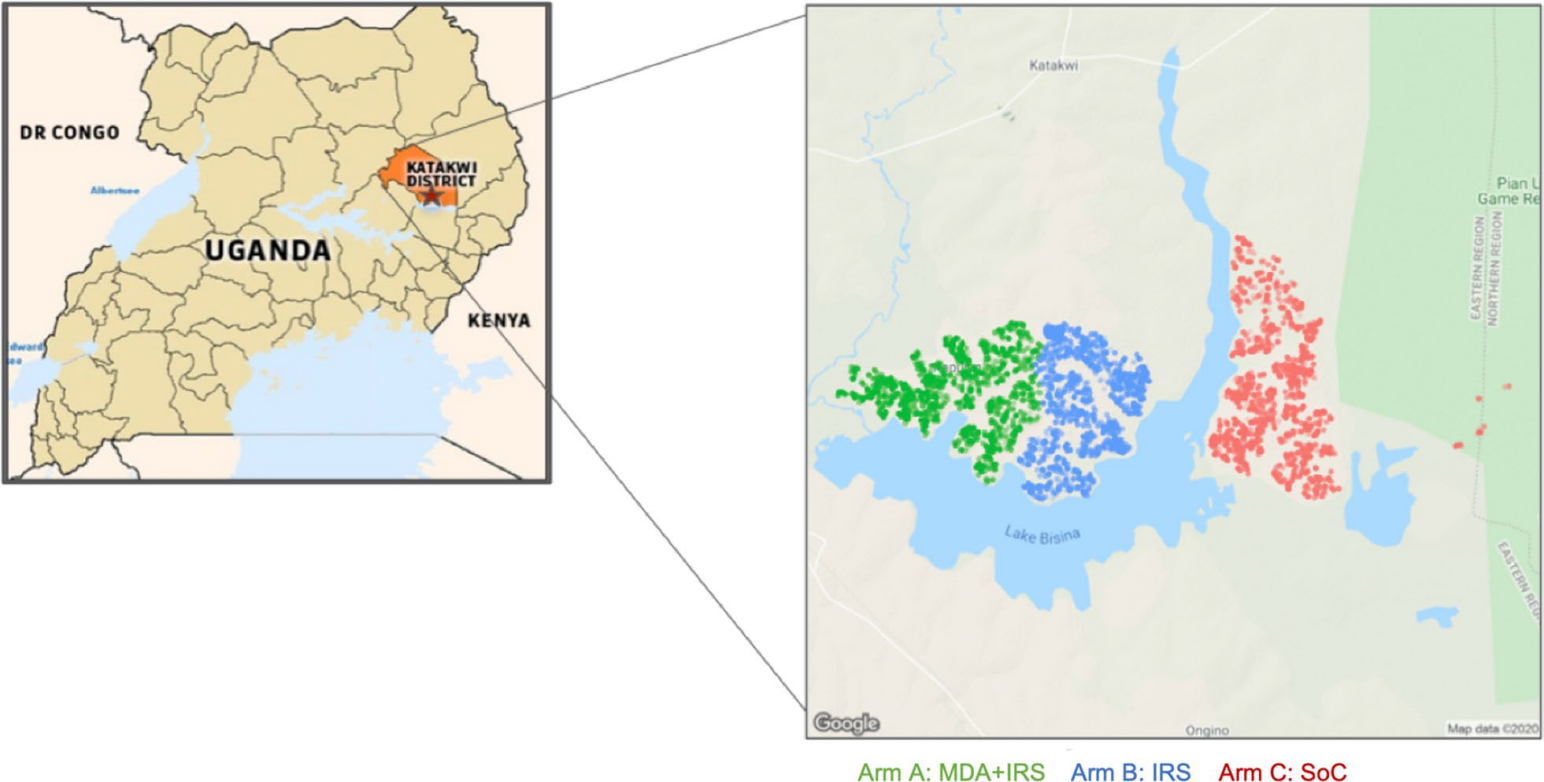
The study site with mapped households and arm assignments. Katakwi district on a map of Uganda, with an inset map showing the study area bordering Lake Bisina. The points on the inset map show geolocated households in 85 villages, color coded by adjacent intervention arms: green for Arm A with MDA + IRS, blue for Arm B with IRS, red for Arm C, standard of care. All sites have ITNs, case management, and IPTp which constitutes standard of care

**Fig. 2:**
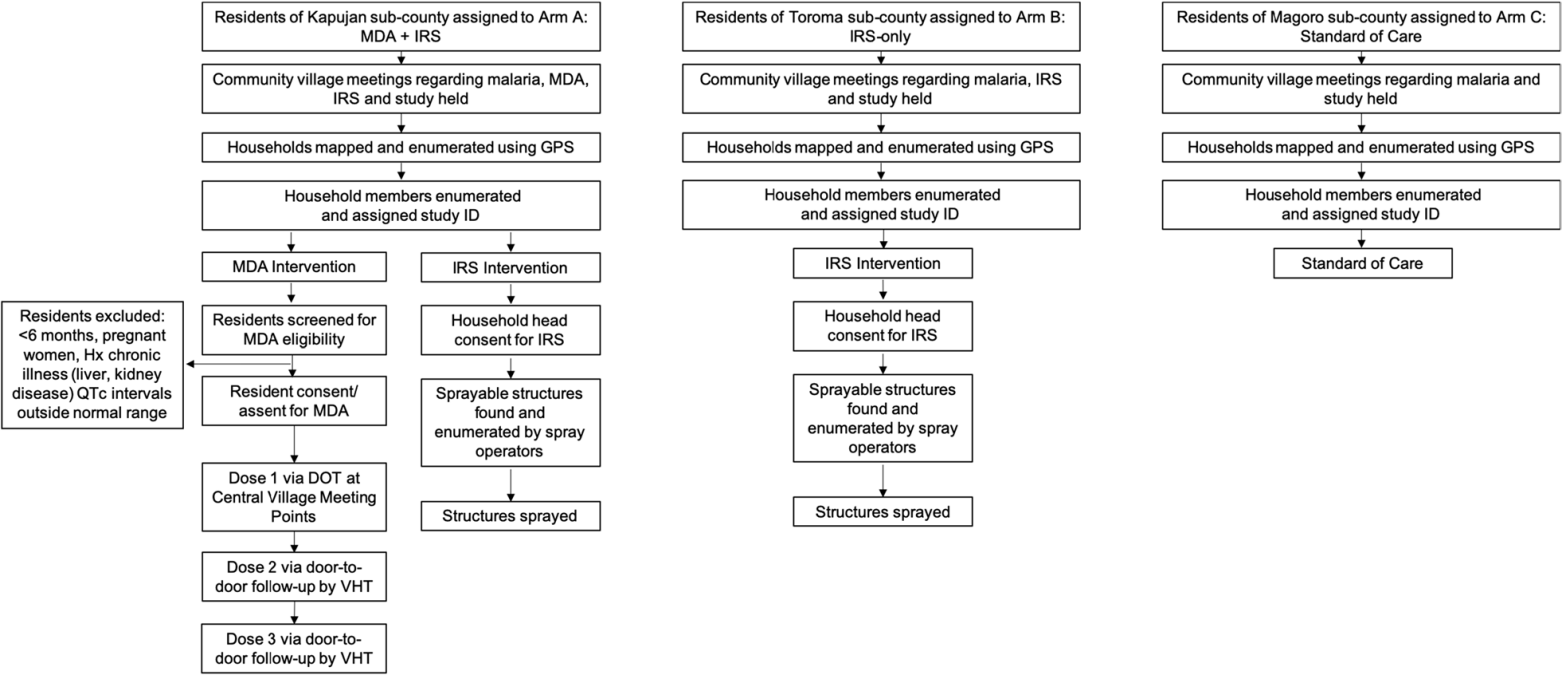
Flow chart of study intervention implementation

The three contiguous sub-counties, shown in Fig. 1, were assigned to the three study arms in the following non-random manner: MDA+IRS was assigned arbitrarily to the sub-county with the highest number of reported malaria cases in 2014, though this was not the sub-county with the highest number of cases in 2016, and the neighboring sub-county was assigned to IRS for operational convenience. At initial enumeration the residential population of the sub-counties varied between 15,738 in the MDA + IRS arm, 10,503 in the IRS arm, and 20,524 in the SOC arm, making an effective 1.5:1:2 allocation. Sub-counties were used as arms not for allocation reasons, however, but because IRS spray in Uganda follows local administrative structure, and is politically complex to administer within arbitrary geographic boundaries. Each arm received one of the following three malaria control packages:

- Arm A (Kapujan sub county): 4 rounds of MDA with DP (Eurartesim) + IRS with pirimiphos methyl (Actellic) + SOC.
- Arm B (Toroma sub county): 4 rounds of IRS with pirimiphos methyl (Actellic) + SOC.
- Arm C (Magoro sub county): SOC only, including ITNs distributed through universal coverage campaign, case management at facilities, and intermittent treatment of pregnant women IPTp.

### Study participants: eligibility and consent

All residents in the three study sub-counties were assessed for inclusion in the study. An enumeration and consenting visit was conducted in all three arms prior to study start. In both intervention arms, an additional screening and consenting visit was conducted before each intervention round, to assess continued eligibility for receiving the intervention. Eligibility and exclusion criteria differed slightly by arm. In the SOC arm, all residents were eligible, no screening was needed, written consent was sought for participation in the study, and all consenting participants were enrolled and assigned unique identification numbers. All residents in the IRS and MDA + IRS arms were eligible for IRS, screened for possible adverse reactions to the insecticides, and written consent was obtained from household heads on behalf of the family; all household members of consenting households were assigned unique identification numbers. All household residents in the MDA + IRS arm were additionally screened for MDA intervention eligibility. Exclusion criteria included: younger than 6 months, pregnant, current symptoms of severe malaria, reported allergies to DP, history of cardiac problems or fainting, family history of long QT syndrome, or currently taking medications known to prolong the QT interval (e.g. antiretrovirals). Eligible adults provided written consent for themselves and any minor children, and children between 8 and 18 also provided written assents. Consented participants in this arm were given unique identification cards that included name, geographical location and demographic information as well unique study ID, and requested to present them at the MDA site at time of treatment. After the first round, barcode scanners were used to confirm the identity, consent and screening status of each participant. Prior to each round, eligibility and consent for MDA+IRS was reassessed. At the MDA treatment sites, consents were confirmed and current medical eligibility reconfirmed. Mapping, enumeration, screening, consents, assents, and sample frames were updated prior to each of the four rounds in both intervention arms.

### Study procedures

#### Social and behavior change communication

To create awareness and uptake of the intervention, and to obtain high coverage rates for MDA and IRS, numerous social and behavior change communication (SBCC) methods were used, before, during and after implementation. Three initial inception meetings at district level and engagement workshops with key macro-level stakeholders helped obtain buy-in and support for the study. This was followed by village meetings conducted in all 85 villages in the study area prior to study start, (and repeated in selected villages as needed during the study period). Next, separate posters describing both MDA + IRS were created, approved by Ministry of Health, and posted publicly in schools, village meeting sites and other venues. Radio talk shows (5 in total) and radio messages reinforced awareness and disseminated important study information before and throughout the study. Immediately prior to the first round of IRS and MDA, two village health team (VHT) coordinators from each village were equipped with posters and mobilized to deliver door-to-door information on both MDA and IRS to all households in the intervention arms, and before each subsequent round as needed. In addition, a gender-sensitive community advisory board comprised of community leaders was instituted and facilitated to meet (4 times in total), to ensure continued engagement and community ownership for the interventions conducted over the 3-year period, and to discuss community results at the end of the study period.

#### Household mapping, and individual enumeration and screening

All households within the study area were mapped to create a sampling frame for estimating coverage of interventions and subsequent evaluations. Household locations were mapped using hand-held eTrex global positioning system (GPS) receivers (Garmin Ltd., Olathe, KS) and readings were taken from the door of the household, if possible, or from a point that was most representative of the household. Additionally, all household residents living in the mapped households were enumerated, screened if living in the intervention arms, and informed consent for study participation was sought as described above.

#### Indoor residual spraying

Spray operations were performed by the study team in compliance with WHO and national standards. IRS was conducted every 8 months in the IRS and MDA + IRS arms using both Hudson and Semco spray pumps fitted with pressure regulators. Both IRS arms received four co-timed rounds of IRS. Approximately 150 spray operators, 50 washpersons and 13 parish store operators were recruited from the target sub-counties, medically screened (pregnant and nursing persons were excluded), and trained on all spray operations including environmental monitoring and compliance, use, and maintenance of spray equipment and personal protective equipment (PPE). In advance of each spray round, the study team marked eligible households with chalk or pen. All communities were alerted to the spray campaign, spray dates, and special instructions in advance. Each spraying exercise in a given parish of 5–10 villages was conducted within a 10-day period, and the entire spray round was conducted within a month. Both VectorLink Uganda and visiting Innovative Vector Control Consortium (IVCC) East African regional teams provided specialized spray quality intensives and monitoring support. The numbers of houses/structures found and those sprayed by the spray operators were counted per parish and cumulative totals sent by short message service (SMS) on a daily basis.

Mosquito resistance to pirimiphos-methyl and other insecticides was monitored via susceptibility testing, including field rearing of captured larvae and exposure to insecticide. This was performed each year during planned spray activities. Quality control testing for IRS was done by cone bioassay testing of representative walls, with repeated cone bioassays providing information about the residual efficacy and the decay rate of the insecticide.

#### Mass drug administration

All eligible and consented residents in the MDA+IRS arm received a three-dose treatment course of DP within a two week period of IRS implementation. A mix of fixed point campaign style and house-to-house sweeps approach was used to administer DP by MDA. Details of this approach are documented in an earlier publication [49]. In summary, the first dose of DP was administered by direct observation (DOT) from 18 fixed village centers to eligible participants. At these sites, each participant was provided instruction on the MDA process, screened, and weighed. Accurate DP dosing was provided according to weight-based guidelines in 8 weight bands. The second and third doses were given to the participants (in distinctive easy to use packaging) with instructions on how the medication should be taken from home on the second and third day. For participants less than 15 years, instructions were provided to their care-takers on how to administer the medication. To ensure compliance, study VHTs conducted house-to-house follow-up sweeps to check if participants had taken their second and third doses, and asked for the empty blister packs/medication envelopes.

#### Standard of care

All sub counties received ITNs by mass campaign conducted nation-wide by Uganda’s MOH in April 2017. In addition, malaria case management and provision of IPTp was performed in all three arms, according to national guidelines, in 8 facilities located in the study area. The study personnel slightly enhanced the commodity supply chain in the study area by ensuring buffer stocks of rapid diagnostic tests (RDTs), ACTs and intravenous (IV) artesunate to facilities as needed to limit their stock-out. Figure 2 provides a summary of the trial flow progress.

### Evaluation methods

To evaluate the impact of the study interventions on malaria prevalence, cross-sectional community surveys were conducted at baseline and every 5–7 months thereafter, with the exception of survey number three, which was conducted 3 months after the second spray round. The surveys collectively provide a composite picture of the prevalence response to the intervention rounds at the $\sim$6(+ 3) month points post-intervention.

**Fig. 3:**
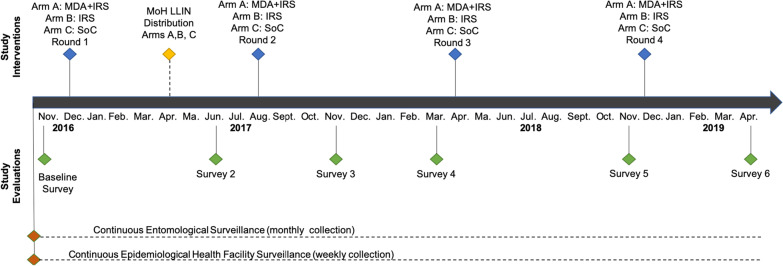
Chronology of the study interventions and monitoring activities. The bottom six green diamonds represent the six cross-sectional surveys in November 2016, June 2017, November 2017, March 2018, November 2018 and April 2019. The four top blue diamonds represent the co-timed intervention rounds (Arms A B) in late November/ early December 2016, August 2017, April 2018, and late November/early December 2018. The top yellow diamond shows the universal ITN coverage campaign conducted in April 2017 by MOH. The bottom two orange diamonds show ongoing entomological and health facility epidemiological surveillance

**Fig. 4:**
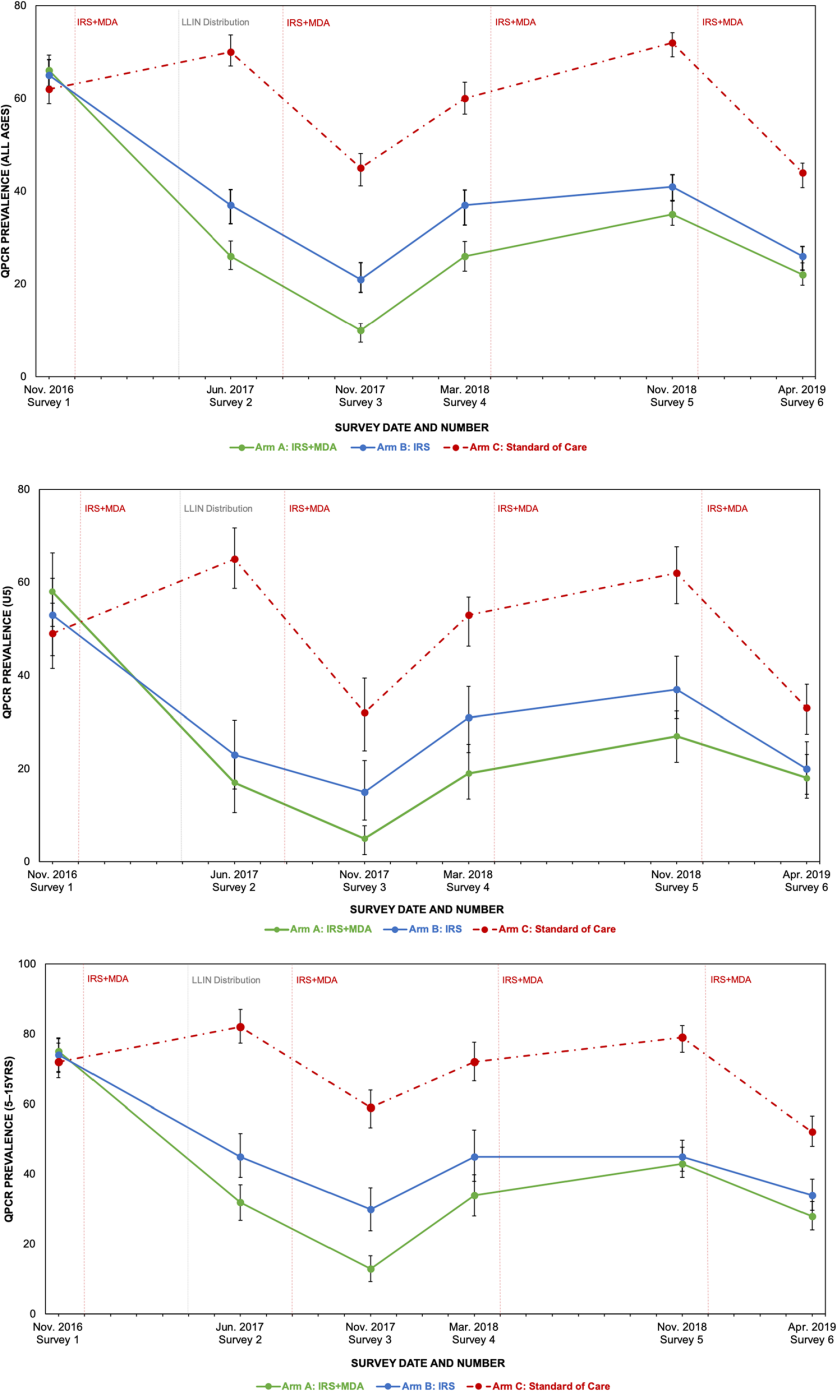
Cross-sectional malaria prevalence by arm, at six time points (all ages, under 5, and children 5–15). The vertical dashed red lines show the co-timed IRS + MDA and IRS only intervention rounds in the IRS arms, while the vertical grey dashed line shows the universal ITN coverage campaign in all three arms. The points show the cross-sectional survey sample percent qPCR positivity at the times of the surveys, while the bars represent the associated 95% confidence intervals for population prevalence

**Fig. 5:**
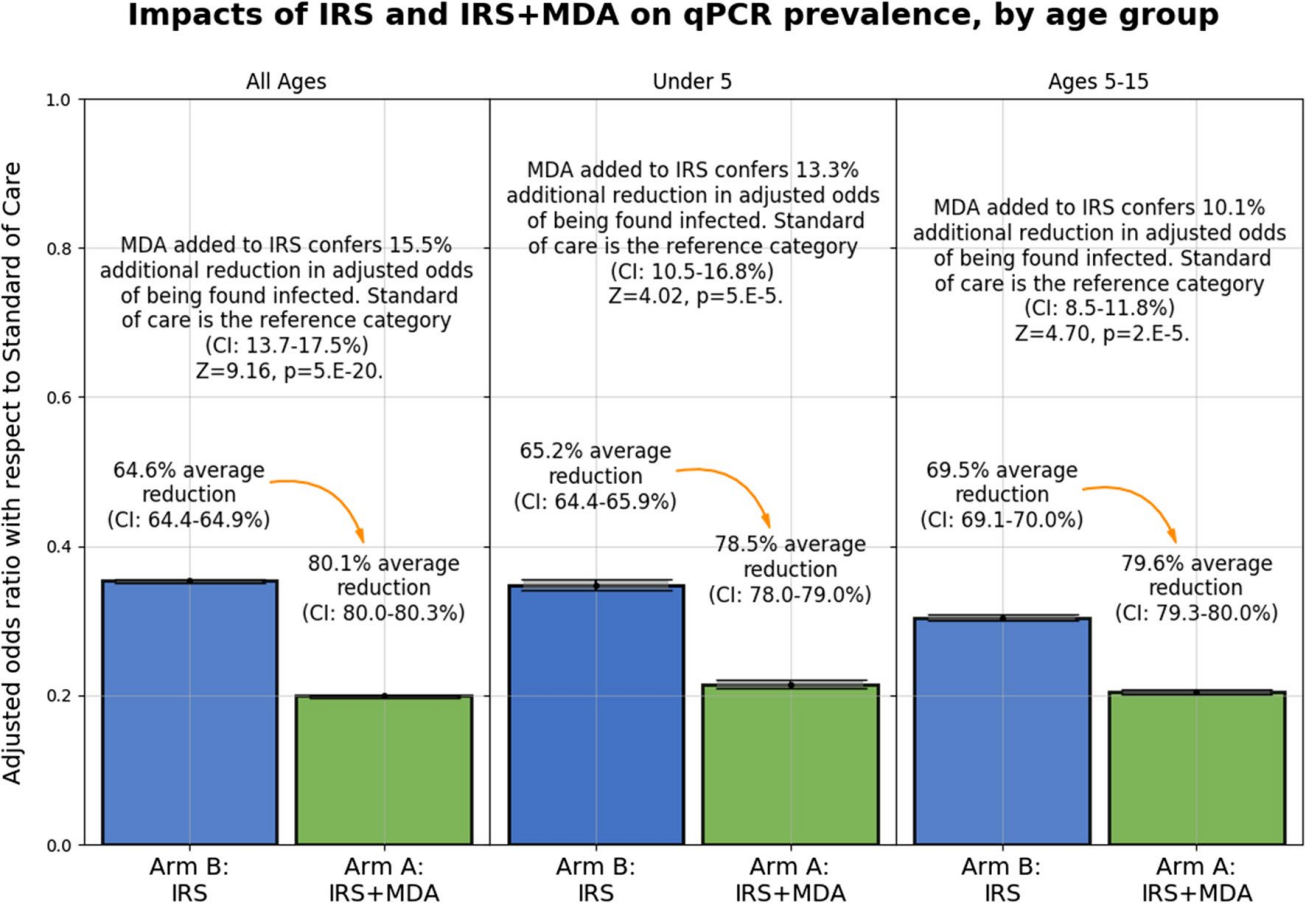
Adjusted odds of being found infected if resident in IRS + MDA or IRS arm relative to resident in standard care (children under 5, children 5–15, and all ages in panels left to right)

Household surveys consisted of three components: (1) a survey questionnaire administered to heads of households, (2) a women’s survey questionnaire, administered to women aged 15–49 years, adopted from the Roll Back Malaria Monitoring and Evaluation Reference Group [50], and (3) a clinical survey including laboratory testing of all household residents present at the time of the survey. Figure 3 shows the timing of the interventions and surveys.

#### Survey sample size and selection

Six surveys were conducted as shown in the timeline of Fig. 3. A sample size of 200 households per arm was chosen; assuming that on average individuals will participate in each household, a sample size of 200 households (n = 800) allows an estimate of the overall parasite rate in each sub-county with a minimum precision (1/2 of the 95% confidence interval) of 0.03. These estimates are conservative as they assume a true parasite rate of 0.5 and a design effect (due to within household clustering of 2). Following round 4 of interventions, the sample size for the community surveys was expanded to 300 households (n = 1200) per sub county. Prevalence in both intervention sub counties had dropped below the expected values, and a sample size of 200 might have been inadequate to distinguish large relative differences between arms. A sample size of 300 households per arm will detect an absolute prevalence difference of +/- 3% at low prevalence levels (e.g. between 3% and 6%, a 100% relative difference), assuming household correlation of 0.2, power of 0.8 and alpha set at 0.05. This yields an effective sample size of 1500 after adjusting for household correlation. This estimate was calculated by first deriving the total population needed for the expected effect size (3%), using a standard sample size equation for a two-sample test of differences in proportions using the pwr package in the R computing software. Then, a standard equation for cluster design effect was applied to estimate the number of households and individuals needed to achieve the required sample size (i.e., the required sample size needs to match the effective sample size after adjustment for correlation among observations from individuals living in the same households). This was done using the following equations,

1 $$\begin{aligned} DE=1+(n-1)p, \end{aligned}$$

where *DE* is the design effect, *n* is the average cluster size (i.e., 4 persons per household assumed),and *p* is the correlation among individuals from the same household (this was 0.2 in previous surveys), and,

2 $$\begin{aligned} ESS= (n*k)/DE, \end{aligned}$$

where *ESS* is the effective sample size and *k* is the number of clusters. A new set of households was randomly selected for each survey. In order to create a non-biased sampling frame, all households in the three sub-counties were enumerated before each survey. Thereafter a computerized number generator using a probability proportion to village size approach was used to randomly select approximately 200 households (for the first four surveys) and 300 households (for the fifth and sixth survey) from each of the sub-counties.

#### Laboratory procedures

The clinical survey component included a finger-prick blood sample for a thick blood smear and for an RDT, measurement of hemoglobin, and preparation of filter paper blood samples for later analysis by quantitative polymerase chain reaction (qPCR). All specimens were barcoded and linked to their corresponding surveys. Thick and thin blood smears were prepared in the field for microscopy. At the laboratory the blood smears were stained with 2% Giemsa for 30 minutes and evaluated for the presence of asexual and sexual parasites (gametocytes). A thick blood smear was considered negative if examination of 100 high-power fields revealed no asexual parasites. For quality control, all slides were read by a second microscopist, and a third reviewer settled any discrepant readings. qPCR was performed on the filter-paper samples. Hemoglobin estimation was carried out on site using a battery-operated portable HemoCue analyzer (HemoCue, Anglom, Sweden). Rapid diagnostic testing was also conducted by the field team using lateral flow assay from SD-Bioline. Filter paper blood samples taken off and stored at $-20^\circ$C before being assessed for parasitemia by qPCR. During qPCR deoxyribonucleic acid (DNA) was extracted using Qiagen spin columns. Parasite DNA was detected using nested PCR targeting the 18S rRNA gene. Parasite density was estimated for all positive PCR samples. Duplex 10 mL reactions amplifying both human ($\beta$-tubulin) and Plasmodium (Met transfer ribonucleic acid (tRNA) gene) targets were run for each sample in a 384-well format ABI qPCR machine, model 7500. Delta Ct values between the two targets were estimated for each sample and the mean delta Ct of duplicate wells normalized to the within-run quantitative standard, comprising the WHO International Standard for *P. falciparum* DNA (IS) and representing 500 parasites/mL. The ratio of parasite density in the sample relative to the IS was then multiplied by 500 parasites/mL to obtain the estimate of parasite density. Any samples negative by qPCR but positive by nested PCR were assigned an arbitrary parasite density value of half the minimum density detected. Participants with a temperature of greater than 37.5$^\circ$C were treated with paracetamol as appropriate and those with a positive malaria RDT and no evidence of severe malaria were treated with artemether-lumefantrine (AL) according to national guidelines. Children and adults with a positive malaria RDT plus any signs of severe disease were referred to the nearest health facility for further evaluation and treatment.

#### Analytical methods

##### Prevalence outcomes

All comparisons of prevalence outcomes followed the intention to treat analysis. Population prevalence for each arm was estimated at each survey time point by qPCR positivity, and reported, with a 95% confidence interval, for three age groups of interest: all ages, children under 5, and children between 5 and 15. The baseline survey results for RDT, microscopy, and qPCR were examined separately and qPCR results subsequently compared with the follow-up surveys in the three age categories.

##### Geostatistical analysis

In order to compare the impact on prevalence for residence in the IRS+MDA arm versus residence in the IRS arm, all qPCR prevalence data from the six cross-sectional surveys in each of the three arms was modeled together using a spatiotemporal Gaussian model with random effects, adjusted for age and seasonality. Model-based geostatistical methods [51] provide a principled likelihood-based approach to carry out spatio-temporal predictive inferences of geo-referenced health outcomes. More importantly, this approach combines all survey data mapped in space and time during the study period, and allows modeling of the residual spatio-temporal variation in disease prevalence unexplained by the measured covariates. This model is described in detail in Additional file 1: Appendix I, but in brief includes estimates for age groups (0–10 years, 10–29 years and 29 years or over); time trends, seasonal effects (high transmission season); baseline effects from the three study arms (control, IRS only and IRS + MDA); intervention effects (MDA only and IRS + MDA) and residual spatiotemporal effects including signal variance, range parameter, nugget and temporal effects. The geospatial analysis allows us to make use of the full information of individual-level data using a joint modelling approach.

##### Difference-in-differences analysis

For comparison, secondary difference-in-differences (DiD) analyses were performed comparing all follow-up survey timepoints with baseline for qPCR. Using SAS 9.4, the model used binomial logistic regression models that compared trends in malaria prevalence in the MDA + IRS arm to trends in malaria prevalence in the IRS arm, and adjusting for potential confounding variables, (age, ITN use, and gender). An interaction term between survey time and arm was used to estimate the DiD. Statistical significance threshold was set at $\alpha =0.05$ using two-tailed tests. Log odds were converted to odds ratios by calculating the exponentiated regression coefficient of the interaction terms in the model. Comparisons between each of the surveys and baseline, and MDA+IRS vs. IRS are presented, and additional analysis details and results are provided in Additional file 1: Appendix II.

## Results

### Participant characteristics

A total of 46,765 individuals and 8004 households across all three study arms were enumerated at baseline. Roughly 51% of the population were females, and 16% were children under 5 years of age. Table 1 shows study population demography enumerated through household visits prior to the baseline survey.

**Table 1:** Baseline populations and qPCR malaria prevalence by study arm and age category

Population and baseline qPCR prevalence by age	Study arms		
	Arm A: MDA+IRS	Arm B: IRS	Arm C: SoC
Total enumerated population	15,738	10,503	20,524
Total children under 5 years (U5), %	2551 (16.2)	1606 (15.3)	3501 (17.1)
Total children 5–15 years, %	5666 (36.0)	3585 (34.1)	7169 (34.9)
All ages			
Sampled population	807	809	912
qPCR +/n^a^	514/779	510/785	520/839
Malaria prevalence, % (95% CI)	66.0 (62.6–69.3)	65.0 (61.6–68.3)	62.0 (58.9–65.3)
U5			
Sampled population	155	141	213
qPCR +/n	87/149	72/137	95/196
Malaria Prevalence, % (95% CI)	58.4 (50.5–66.3)	52.6 (44.2–60.9)	48.5 (41.5–55.5)
5–15 Years			
Sampled population	319	323	348
qPCR +/n	231/312	234/317	229/316
Malaria prevalence, % (95% CI)	75.0 (69.2–78.9)	73.8 (69.0–78.7)	72.5 (67.5–77.4)

^a^qPCR results missing, n = 125

**Table 2:** SMS/Sprayer operator-based IRS coverage: houses sprayed/houses found

Intervention round and characteristic	Study arms		
	Arm A: MDA+IRS	Arm B: IRS	Total
Round 1: Dec, 2016			
Houses sprayed	6483	5179	11,662
Houses found	6509	5339	11,852
Coverage (%)	99.6	97.0	98.4
Population protected			30,741
Round 2: Aug, 2017			
Houses sprayed	6173	5429	11,602
Houses found	6235	5597	11,827
Coverage (%)	99.0	97.0	98.1
Population protected			31,663
Round 3: Apr, 2018			
Houses sprayed	6658	5541	12,199
Houses found	6718	5712	12,435
Coverage (%)	99.1	97.0	98.1
Population protected			32,979
Round 4: Dec, 2018			
Houses sprayed	6355	5280	11,635
Houses found	6426	5399	11,824
Coverage (%)	98.9	97.8	98.4
Population protected			32,438

**Table 3:** MDA coverage by method and round (Arm A), December 2016–December 2018.

Coverage by dose and method	Round (month, year)			
	Round 1, Dec 2016 n (%)	Round 2, Aug 2017 n (%)	Round 3, Apr 2018 n (%)	Round 4, Dec 2018 n (%)
Dose 1				
Fixed distribution	12,536 (80.0)	12,586 (83.0)	12,366 (80.0)	12,449 (80.2)
Dose 2				
Door-to-door monitoring	NA	12,412 (81.8)	12,344 (79.8)	12,399 (79.9)
Dose 3				
Door-to-door monitoring	NA	12,408 (81.8)	12,343 (79.8)	12,399 (79.9)
Total (N)	15,668	15,170	15,460	15,525

### Baseline prevalence

The initial survey was performed in November 2016, before the first IRS and MDA rounds in December 2016. All arms were found to be highly infected at baseline, with all ages qPCR prevalence of 62–66%, and between 73–75% in children ages 5–15 (Table 1). Described further in Additional file 2: Appendix III, microscopy prevalence was between 21–26%, with the MDA + IRS arm most highly infected and the SOC arm least infected. In contrast, RDT prevalence was between 36–47%, and showed the opposite trend, with the MDA + IRS arm least infected and the SOC arm most infected. The qPCR trends tracked the microscopy trends more closely than they tracked the RDT trends, but were considerably more sensitive than either, and showed less overall variation between arms than did either the microscopy or the RDT. Baseline malaria prevalence by age group and study arm is summarized in Additional file 2: Appendix III.

### Intervention coverage

#### IRS coverage

IRS coverage in both arms in all 4 rounds was over 97% of structures sprayed over structures found. This method of measuring coverage can be inaccurate when used for targeted spray operations by spray operators who are unfamiliar with the area they are spraying; however in this study, spray operators sprayed their own villages and neighboring villages, and all houses in the designated area were mapped, enumerated and targeted for spraying, which created positive social pressure not to miss compounds. Due to strong SBCC programs prior to spraying, almost all residents were home with belongings already prepared outside their home when spray operators arrived. As noted previously, IRS was welcomed by the community [52] and very few homes were found locked. Table 2 contains household information on structures sprayed by intervention arm.

#### MDA coverage

High MDA compliance was the main goal for the SBCC programs, and the investment in these activities was robust, with high community participation resulting. The denominator for MDA coverage included all persons normally resident in the study site, whether eligible or not. For rounds 1,3 and 4, MDA directly observed first dose coverage was approximately 80% or more, with a 1% fall off for second and third dose compliance ascertained by blister pack and verbal confirmation (See Table 3). Round 2 MDA coverage was slightly higher at 83% for directly observed first dose, and 82.2% after second and third dose compliance.

#### Cross-sectional surveys and qPCR testing

Primary analysis was based on qPCR results, because the qPCR and microscopy results proved more consistent across arms than RDT findings, and because qPCR was more sensitive throughout than either microscopy or RDT (see microscopy and RDT results in Additional file 2: Appendix III). qPCR results at baseline were more similar across arms, with smaller relative dispersion, than those from microscopy. During the six cross-sectional community surveys, 15925 qPCR tests for *P. falciparum* were conducted, of which 6730 (42.3%) were positive. Cross-sectional qPCR prevalence results by survey, number, date and intervention arm are presented for different age categories for all 6 surveys in Fig. 4.

#### Differential impact on malaria qPCR positivity of IRS over standard of care, and of MDA+IRS over IRS, measured over all surveys: geospatial analysis

**Fig. 6:**
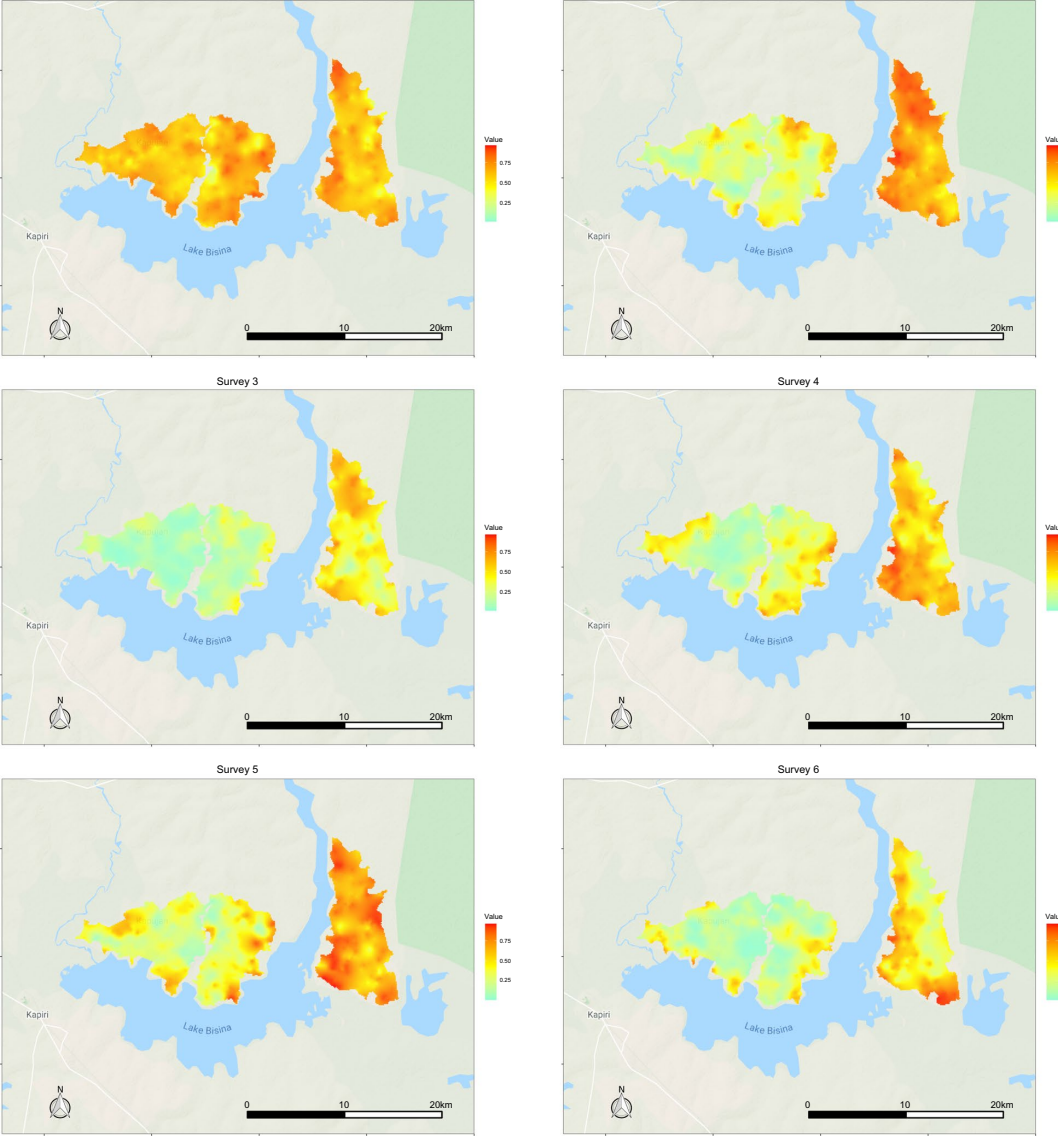
Heat maps of predicted spatial heterogeneity in malaria prevalence in children under 5 in surveys 1–6

**Fig. 7:**
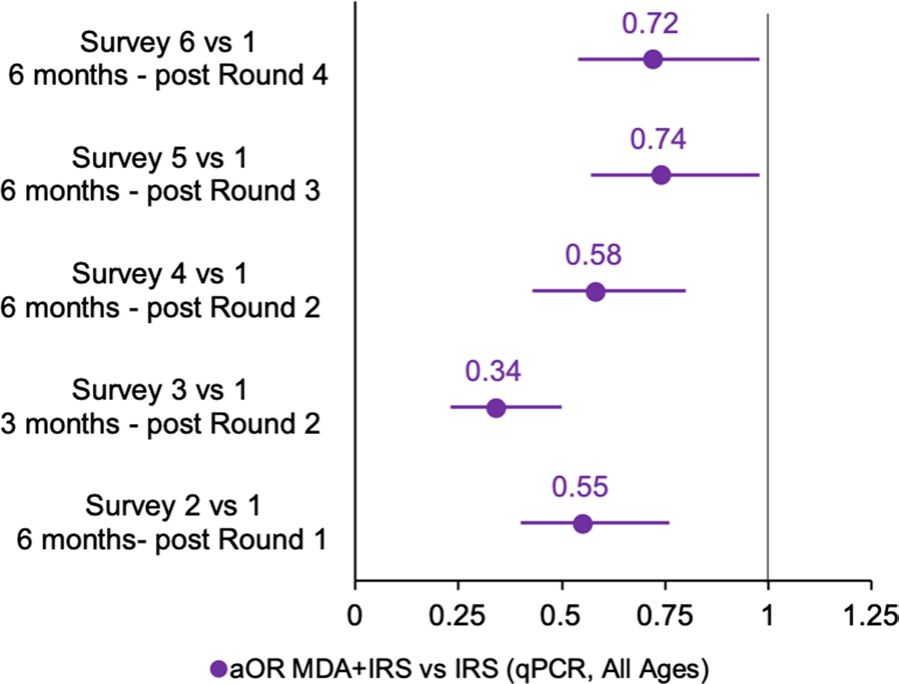
DiD adjusted odds ratio of qPCR confirmed malaria (all ages), MDA + IRS vs IRS, by survey timing compared to baseline

The differential impact of IRS over standard care, and of MDA+IRS over IRS alone, over all 6 surveys, was analyzed using a geostatistical model with results summarized in Fig. 5. Residence in the IRS arm was associated with a 70% reduction in odds of being found infected relative to the standard care arm for children of age 5–15, and a 65% reduction for all ages as well as for children under 5. A resident of any age in the arm with both IRS and MDA had an average 80% decrease in odds of being found infected as compared with a person under the same conditions living in the standard care arm. This result was consistent across age groups, with children under 5 and 5–15 resident in the MDA+IRS arm having a 79% and 80% decrease, respectively. Comparing the MDA+IRS and IRS only arms, we estimate a significant additional 15.5% (95% CI: [13.4%, 17.9%], p = 5e−20) decrease in the adjusted odds ratio (aOR). For children under 5 and children 5–15 residence in the MDA+IRS arm was also associated with significant additional protection over IRS only, measured on average 5–7 months post-campaign. For children 5–15: aOR reduced 13.3% (95% CI: [10.5–16.7%], p = 5e−5) and for children under 5: aOR reduced 10.1% (95% CI: [8.5–11.8%], p = 2e−5). The geostatistical model estimated the temporal and spatial correlation for the qPCR results based on the date and geolocation of each sample. The estimated range of the spatial correlation was found to be $\sim$1.0 km, beyond which the spatial correlation took on values smaller than 0.05. Figure 6 shows the predicted geography of malaria prevalence, including non-surveyed locations, for children under 5 at the six survey time points of Fig. 3.

#### Differential impact of MDA + IRS over IRS on malaria qPCR positivity measured after each survey: DiD analyses

Figure 7 summarizes results from a secondary DiD analysis comparing the change in the MDA + IRS arm to the change in the IRS arm at different survey points from baseline. The aOR of qPCR malaria confirmation was 45% lower in residents who received one round of MDA + IRS compared to the IRS arm (DiD aOR = 0.55, 95% CI: [0.40, 0.76], p < 0.001), and remained consistent when measured 6 months following round 2 (aOR = 0.58, 95% CI: [0.43, 0.80], p < 0.001). When measured 3 months following round 2, the adjusted odds of qPCR malaria infection in residents who received MDA + IRS were 66% lower than residents who received IRS, (aOR = 0.34, 95% CI: [0.23, 0.50], p < 0.001). Six months following rounds 3 and 4, the adjusted odds of qPCR malaria confirmation were not as low: (aOR = 0.74, 95% CI: [0.57, 0.98], p < 0.05) and (aOR 0.72, 95% CI: [0.54, 0.98], p < 0.05) respectively. Details of this analysis and these comparisons may be found in Additional file  1: Appendix II.

#### Adverse events

Both adverse events (AEs) and serious adverse events (SAEs) were monitored by the study team and SAEs reported in writing to Sigma Tau as well as to University of Makerere’s internal review board (IRB) and the study’s project advisory committee (PAC), a subset of whose members functioned as a data safety and monitoring board (DSMB). Three SAE’s were reported in the MDA arm. The only one with a high likelihood of being related to the study medication was a case of toxic epidermal necrolysis in a two year old girl who fully recovered. One study-related adverse event of skin rash from prolonged sleeping contact with a newly sprayed wall was reported in the IRS only arm. The leading AEs after MDA were lethargy and stomach upset.

## Discussion

Following interest in the potential of chemoprevention as a control tool [20, 21], recent WHO guidelines for malaria contain a conditional recommendation for MDA for burden reduction in moderate to high transmission environments. However, very low certainty evidence shows MDA impact on prevalence in high transmission [33, 34]. Modeling and experimental work suggest that the co-timed use of MDA with vector interventions could be a particularly effective high burden control tool, as the concurrent IRS extends protection against re-infection to times well beyond initial chemoprophylaxis [35, 36]. This study examines the impact of co-timed MDA+IRS versus IRS, and of MDA + IRS and IRS versus standard of care, on population malaria prevalence at long time points (5–7 months) past a single MDA round, in a high transmission context, in order to test the modelled predictions of synergy.

Within the limitations of the quasi-experimental design and the single large cluster used per arm, this study demonstrates a significant, surprising advantage for co-timed MDA and IRS over IRS alone, and strongly suggests that further investigation should be pursued. Visual inspection of measured qPCR results over all 6 surveys, depicted in Fig. 4, shows that every age category was highly infected at baseline in November 2016, especially children 5–15, who have been shown to account for more than half of the infectious reservoir in a similar transmission environment in Uganda [53]. After interventions, the arms clearly separate. The SOC arm, which like all the others received new ITNs in April 2017, is consistently higher than the others, with seasonal and/or annual fluctuations. Some possible intervention impact in the SOC arm, presumably from the ITN distribution, is seen at Survey 3. Both IRS arms measured much lower prevalence than SOC at all surveys post-baseline, while the MDA + IRS arm consistently contained fewer infected individuals than the IRS arm. The differential gains from MDA+IRS over IRS at the single survey that measured prevalence 3 months after the round, Survey 3, are particularly striking when compared with the seasonally equivalent baseline: in children under 5, qPCR positivity dropped more than an order of magnitude, from 58% at baseline to 5% one year later. By comparison, positivity for children under 5 in the IRS-only arm dropped from 53% to 15% (Fig. 4), a factor of roughly 3.5. Additional tables of malaria prevalence by study arm, age and diagnostic tool can be found in Additional file 2: Appendix III.

Geostatistical modeling analysis using all qPCR data from all six cross-sectional surveys and an intention to treat approach showed that, over the 32 months of the study after baseline, residents of the IRS arm were $\sim$65% less likely to be found infected with malaria than those living in the standard of care arm with universal ITN coverage only, a large preventative difference. These results highlight the strong additional protection in this high transmission setting provided by an IRS campaign over ITNs alone. Significant additional protection was observed when a co-timed MDA campaign was included, as residents in the MDA+IRS arm were $\sim$80% less likely to be found infected than those resident in the standard of care arm, and had a 15.5% (95% CI: [13.4%, 17.9%], p = 5e−20) decrease in the adjusted odds ratio as compared with those resident in the IRS only arm, measured roughly 6 months after the campaigns. For child populations, residence in the MDA + IRS arm was also associated with significant additional protection over the IRS-only arm. For children 5–15: aOR reduced 13.3% (95% CI: [10.5–16.7%], p = 5e−5) and for children under 5: aOR reduced 10.1% (95% CI: [8.5–11.8%], p = 2e−5) (Fig. 5).

Secondary DiD analysis, also intention to treat, compared single prevalence survey timepoints to baseline, and confirmed the significant protective impact of MDA+IRS over IRS alone (Fig. 7). These comparisons showed significantly reduced aOR for the MDA + IRS arm compared with the IRS, with regression coefficients ranging from 0.28 to 0.66 for surveys 2–6. MDA + IRS appears even more protective when compared with IRS by DiD analysis than by geospatial modeling, but the DiD analysis involves a more limited selection of the data, because it does not make use of the full qPCR dataset, but only considers data from pairs of groups separately or in an aggregated format; therefore, the geospatial analysis is presented as primary. The smallest protective advantage for MDA+IRS over IRS was noted in survey six (an expected seasonal low point), compared to survey one (an expected seasonal high point). Survey three, on the other hand, is an expected seasonal high point, conducted at the same time of year as the baseline survey, but because it was conducted only 3 months rather than 6 months after the MDA round, provided an alternate window into the dynamic preventive effect of the MDA. Additional DiD comparisons between MDA+IRS and SOC and between IRS and SOC can be found in Additional file 1: Appendix II.

It’s striking that survey data, measured 5–7 months after a single treatment round of MDA in this high transmission environment, still show significant protective advantage from MDA + IRS over IRS. However, cross-sectional measurements of prevalence would almost certainly be lower and even more differentiated at points in time closer to the MDA round. High-coverage MDA immediately lowers population prevalence which then slowly rebounds, as chemoprophylaxis expires and re-infection occurs. Under the assumption of ideal DP protection and compliance with medication regimen, in the first month after the intervention, $\sim$79% of the population presumably enjoyed protection through the $\sim$28 day chemoprophylactic period of DP [54]. Survey three, performed 3 months after the round, showed the lowest prevalence captured in the MDA+IRS arm, and also a significantly more pronounced differential impact for the co-timed MDA, with 66% reduced aOR of infection for residents in the MDA+IRS arm compared with residents in the IRS arm, as opposed to reductions of 28–45% for other survey times points (see Fig. 7). This protection waned steadily in the months following the MDA round, as treated individuals became reinfected. Survey four, taken several months later, indicate the resurgent return of parasitaemia in the study area as expected.

Sustainability is often a concern for MDA in a control context, but absent a change to the underlying forces of infection, resurgence will reliably occur when any control intervention ceases [55]. Effective interventions are also associated with a rapid shift in the remaining burden of malaria from younger people to older people, an advantage not captured in burden data alone [56].

Because prevalence in most of the surveys was measured at 5–7 months after the MDA, the deepest impacts of MDA almost certainly occurred outside the survey observation window. Health facility catchment areas are not constrained, so it is difficult to reliably quantify this effect through facility based case surveillance trends. Monthly cross-sectional surveys following an MDA + IRS and an IRS intervention, or a cohort study of incidence following the interventions, could help capture the full protective impact of MDA+IRS. As IRS costs much more than MDA per person, the addition of MDA to IRS is likely to be cost effective, especially as the benefits of the combination are expected to include reduction of clinical cases in the first months after the joint round.

## Conclusion

This trial showed that a significant protective impact of MDA was sustained for long times (5–7 months) after a treatment round, a surprising result in high transmission. The 2013 Cochrane review of MDA explicitly evaluated studies for sustained impact beyond 6 months, finding only a few low transmission trials that met this criterion [57, 58]. Similar findings from a large comparative modelling study suggested that MDA (administered alone, and not co-timed with vector control) was much less likely to have durable impact in high transmission [59]. This trial’s finding is also in distinction to other trials of MDA impact in similar settings [40], and to cohort studies of individual treatment in absence of vector control [37]. The results suggest that MDA impact in this high transmission setting was indeed protracted by co-timed vector control as predicted by modelling.

Limitations of this study include the fact that population-based interventions were assigned without blinding or randomization. Although qPCR prevalence in all three arms was similar at baseline in 2016, the MDA + IRS arm was arbitrarily assigned to the sub-county with the highest number of clinical cases in 2014 when the study was first conceived. Performing targeted sub-district level IRS had not been practiced in Uganda at the time, and bureaucratic reasons dictated early, clinically justified identification of the sub-counties receiving IRS. Due to prohibitive cost constraints in creating large-scale IRS clusters, and the concern of contamination through mosquito flight at smaller scales (for instance, at village level), a single large community intervention cluster (an entire subcounty, with 18–30 villages) was used for each arm. As equipoise precluded the establishment of a control arm without any malaria prevention in place, and also the use of MDA without IRS, it is not possible to separately distinguish the effect of ITNs vs no control or to evaluate the impact of MDA in the absence of IRS. Only the first dose of MDA was observed, with the second and third dose inferred from collection of empty blister packs. If compliance was imperfect, MDA coverage would be lower than assumed, which would reduce the additional impact of MDA. Intervention impact on prevalence, with one exception, was measured at timepoints 5–7 months from each of the four rounds of intervention; these measures are unable to provide a comprehensive window into the population response to the intervention at shorter intervening time periods.

As previously reported, campaign-style all-ages MDA was both easy to implement [49] and highly acceptable in this high transmission environment [52], and achieved high coverage of $\sim$79% for all four rounds. IRS for vector control requires extensive community engagement and mobilization just as MDA does, and the two activities probably benefited from joint investment in SBCC activities and joint momentum. MDA’s possible contribution to antimalarial resistance [60] is a valid concern, but in Uganda this is mitigated by the use of DP for MDA while artemether-lumefantrine (AL) is used for frontline therapy; the observed counter-selection between DP and AL [61–63] may offer protection against the development of resistance.

Many high burden countries after the pandemic are seeing large rises in case numbers. To continue to make global gains against malaria, it’s necessary to optimize and target tools for burden reduction in high transmission areas. Vector control is still the mainstay of prevention, but chemoprevention strategies are an important additional tool for burden reduction in high transmission settings, as noted by the new WHO conditional recommendation [33]. The co-timing synergy offers an especially effective way to employ MDA for burden control. Increased control impact from careful co-timing of MDA together with vector control is predicted for all forms of vector control and has been modeled for MDA + ITNs as well as MDA + IRS [35]. Because vector control is usually delivered less frequently than chemoprevention, a co-timed use of MDA represents a low-frequency use of chemoprevention, and though maintenance and continuity are always needed in control settings, the reapplication demands and associated costs are modest. In highly infected communities, an annual round of MDA does not represent additional drug pressure on a community, but instead a demographic reorganization (as most treated clinical infections are in children) and synchronization of treatment. Co-timed IRS+MDA may be an effective, practical and acceptable intervention for sustained burden reduction in high burden settings. Further investigation of the impact of co-timed MDA + IRS in highly infected communities, including cohort studies of incidence following intervention, is strongly suggested.

## Supplementary Information

**Additional file 1. Appendix I:** Spatiotemporal geostatistical model; **Appendix II:** Additional DiD analyses.

**Additional file 2. Appendix III:** Malaria prevalence tables.

## Data Availability

The datasets supporting the conclusions of this article are available in the figshare repository, https://figshare.com/s/0dda5516aafbda8bea48.

## Abbreviations

ACT: Artemisinin combination therapy
AL: Artemether-lumefantrine
aOR: Adjusted odds ratio
CI: Confidence interval
DHIS2: District Health Information System Version 2
DiD: Difference-in-differences
DOT: Directly observed therapy
DP: Dihydroartemisinin-piperaquine
DNA: Deoxyribonucleic acid
DSMB: Data safety and monitoring board
GPS: Global positioning system
IPTp: Intermittent preventive treatment for pregnant women
IPTsc: Intermittent preventive treatment in schoolchildren
IRB: Internal review board
IRS: Indoor residual spraying
ITN: Insecticide treated net
IV: Intravenous
IVCC: Innovative Vector Control Consortium
MDA: Mass drug administration
MOH: Ministry of Health
PAC: Project advisory committee
PMI: President’s Malaria Initiative
PPE: Personal protective equipment
qPCR: Quantitative polymerase chain reaction
RDT: Rapid diagnostic test
SAE: Serious adverse events
SBCC: Social and behavior change communication
SMS: Short message service
SOC: Standard of care
tRNA: Transfer ribonucleic acid
U5: Under 5
VHT: Village health team
WHO: World Health Organization
WHOPES: World Health Organization Pesticide Evaluation Scheme
