# Supplementary material for: Impact of population based indoor residual spraying with and without mass drug administration with dihydroartemisinin-piperaquine on malaria prevalence in a high transmission setting: a quasi-experimental controlled before-and-after trial in northeastern Uganda
Source: BMC Infect Dis. 2023 Feb 6;23:72. doi: 10.1186/s12879-023-07991-w (PMC9901833; doi:10.1186/s12879-023-07991-w)
Supplement: Supplementary file 1 — Additional file 1. Appendix I: Spatiotemporal geostatistical model; Appendix II: Additional DiD analyses. [file 12879_2023_7991_MOESM1_ESM.pdf]

SUPPLEMENTARY INFORMATION

Supplementary Information: Impact of population based indoor residual spraying with and without mass drug administration with dihydroartemisinin-piperaquine on malaria prevalence in a high transmission setting: a quasi-experimental controlled before-and-after trial in northeastern Uganda

Full list of author information is available at the end of the article

Supporting Information

Appendix I. Spatiotemporal geostatistical model

Let $p_j(x_i, t_i)$ denote the probability of a positive qPCR result from the j-th individual sampled at i-th location x_i and month t_i, where $t_i \in \{1, \ldots, 31\}$, with $t_i = 1$ representing November 2016. Conditionally on a spatio-temporal Gaussian process $S(x_i, t_i)$ and unstructured random effects $Z(x_i, t_i)$, we modeled the probability of positive qPCR test $p_j(x_i, t_i)$ as logit-linear regression given by,

$$\begin{aligned}\log\left\{\frac{p_j(x_i,t_i)}{1-p_j(x_i,t_i)}\right\} &= \beta_0 + s(a_{ij}) + \beta_4 t_i + \beta_5 I(t_i \in HTS) + \\ &\quad \beta_6 I(t_i \in Baseline) + \beta_7 I(t_i \in Kap_{base}) + \beta_8 I(t_i \in Tor_{base}) + \\ &\quad \alpha_1 I(x_i \in Tor) + \alpha_2 I(x_i \in Kap) + S(x_i,t_i) + Z(x_i,t_i), \qquad (1)\end{aligned}$$

where $s(a_{ij})$ is a linear spline expressed by,

$$\begin{aligned}s(a_{ij}) &= \beta_1 \min(a_{ij}, a_0) + \beta_2 I(a_{ij} > a_1)\min(a_{ij} - a_1, a_1 - a_0) + \\ &\quad \beta_3 \max(a_{ij} - a_1, 0). \qquad (2)\end{aligned}$$

In this expression, a_{ij} denotes the age of person j at location x_i, and $a_0 = 10$ and $a_1 = 29$ are ages where a change in the slope of the linear spline is imposed. The values of 10 and 29 years were chosen based on a deviance profile of all possible combinations for a_0 and a_1 using a standard generalized linear model. In the above equation, β_1, β_2 and β_3 are the slopes of age in different age groups, that is the

effects of age on the log-odds of prevalence in the age groups $0-10$, $10-29$, and $30+$ respectively.

We used linear functions of time (corresponding to β_4) to account for long-term trends. Seasonality was included by classifying the year into a high transmission season (June-August), with prefactor β_5 and low transmission season otherwise.

We accounted for differences in prevalence between different trial arms at baseline by including indicator variables of whether or not a data point was sampled at baseline in ($Baseline$), Kapujan during baseline (Kap_{base}) and Toroma during baseline (Tor_{base}) corresponding to $\beta_6\,\beta_7, \beta_8$, respectively.

To account for the different intervention strategies, Tor represents Toroma, Arm B, which received IRS only while Kap represents Arm A, Kapujan, which received both IRS and MDA. The main regression parameters of interest are therefore α_1, α_2, respectively, which when lesser, represents a greater impact of the corresponding intervention. Arm C, Magoro, the SOC arm, is the reference category.

Table 1 Estimates of the geostatistical parameters.

Variable	Parameter	Point estimate	95% CI
Other covariates effects			
Intercept (Control, low trans.)	$\exp(\beta_0)$	0.4234	(0.3405, 0.5264)
Age trend in $0-10$ y/o	$\exp(\beta_1)$	1.2006	(1.1806, 1.2210)
Age trend in $10-29$ y/o	$\exp(\beta_2)$	0.9368	(0.9288, 0.9448)
Age trend in ≥ 29 y/o	$\exp(\beta_3)$	0.9913	(0.9862, 0.9965)
Time trend	$\exp(\beta_4)$	1.0107	(1.0029, 1.0187)
Seasonal effect (High trans. season)	$\exp(\beta_5)$	1.7408	(1.4697, 2.0619)
Baseline main effect	$\exp(\beta_6)$	1.6108	(1.2547, 2.0680)
Baseline effect in the arm with IRS Only	$\exp(\beta_7)$	2.5774	(1.9520, 3.4033)
Baseline effect in the arm with MDA + IRS	$\exp(\beta_8)$	5.4293	(4.0901, 7.2069)
Interventions effects			
IRS Only	$\exp(\alpha_1)$	0.3533	(0.3150, 0.3963)
MDA + IRS	$\exp(\alpha_2)$	0.1987	(0.1768, 0.2233)
Residual spatiotemporal effects			
Signal variance	σ^2	5.4445	(4.8036, 6.1710)
Range parameter	ϕ	1.0260	(0.8526, 1.2347)
Nugget effect	τ^2	0.4559	(0.3611, 0.5757)
Temporal effect	ψ	2.3645	(1.5550, 3.5954)

We modelled the spatially and temporally correlated random variations $S(x_i, t_i)$ as a stationary and isotropic Gaussian process with the separable correlation function,

$$\rho(u, v; (\sigma^2, \phi, \psi)) = \rho_1(u; (\sigma^2, \phi))\rho_2(v; \psi), \tag{3}$$

where the spatial component of the correlation function is modelled as $\rho_1(u; (\sigma^2, \phi)) = \exp(-u/\phi)$, where ϕ regulates the pace at which the spatial correlation decays for increasing distance u between any two locations, and the temporal correlation function as $\rho_2(v; \psi) = 1/(1 + v/\psi)$ with v being the difference between any two time points. We modelled the spatially unstructured random effects $Z(x_i, t_i)$ as independent and identically distributed Gaussian random variables with variance τ^2.

The final data consisted of 15925 qPCR tests for *P. falciparum*, of which 6730 (42.3%) were positive. Table 1 shows parameter estimates of the geostatistical model of equations 1-2. As expected, the odds ratio increases sharply in the early years of life until age 10, and it decreases moderately thereafter until around the age of 30, after which there is very slow decrease in later years (Fig. 1). The estimate range of the spatial correlation is about 1.0km, beyond which the spatial correlation takes values smaller than 0.05. The estimated range of the temporal correlation is about one month. As indicated by the larger estimate of σ^2 than τ^2, the spatial variation dominates substantially the unexplained variation on a scale smaller than the minimum observed distance.

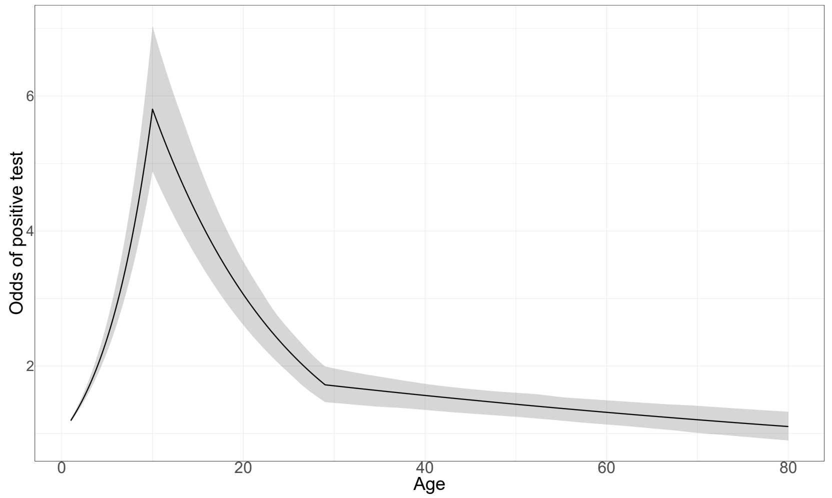

Figure 1 The effect of age on odds ratio. The solid line indicates the odds ratio at different ages, and the shaded grey area is the associated 95% confidence interval.

Appendix II. Additional DiD Analyses

The DiD design is a quasi-experimental method that relies on the assumption that in the absence of any intervention, the study arms receiving the enhanced malaria interventions have identical trends in outcomes as the SOC (control) arm.

Using SAS 9.4, the model used a binomial logistic regression with the proportion of positive malaria cases by diagnostic tool (qPCR, microscopy and RDT) and age

as the dependable variables. Indicator variables for survey timing (baseline/post-intervention surveys) and study arms were used, with co-variates determined through backwards stepwise procedure, and an interaction term between survey timing and study arms. Statistical significance threshold was set at alpha = .05 using two tailed tests. Log odds were converted to odds ratios by calculating the exponentiated regression coefficient of the interaction terms in the model. DiD analyses were not done for positive malaria cases in the 'all ages' group by RDT, since malaria prevalence at baseline was significantly higher in the SoC (Arm C) compared to Arms A and B by this metric.

After adjusting for age, ITN use and gender, Fig. 2 (upper left) shows the odds of qPCR confirmed malaria in residents receiving MDA+IRS were significantly lower compared to residents receiving SOC, at all survey time points. The aOR of qPCR malaria confirmation was 90% lower in residents who received 1 round of MDA+IRS compared to the SoC arm (DiD aOR = .10 [95% CI: .08 –.14], $p < .001$); adjusted odds remained consistent 6 months following rounds 2 and 3 (DiD aOR = .18 [95% CI: .07 – .15], $p < .001$) and (DiD aOR = .17 [95% CI: .13 – .31], $p < .001$), respectively. The odds of malaria 6 months following 4 full rounds of MDA+IRS was 70% lower than the SoC arm (DiD aOR = .30 [95% CI: .23 – .40], $p < .001$).

Residents in the IRS arm (Fig. 2,upper right) also showed significantly lower odds of qPCR confirmed malaria in relation to SoC residents. All surveys measured impact 5 – 7 months following intervention rounds, with the exception of survey 3, which showed significant impact in residents receiving MDA+IRS 3 months after receiving 2 rounds of the intervention compared to residents receiving SoC (DiD aOR = .11 [95% CI: .07 – .15], $p < .001$) and residents receiving IRS (DiD aOR = .30 [95% CI: .21 – .41], $p < .001$). The lower panels of Fig. 2 show the same trends in microscopy confirmed malaria for all ages.

These comparisons highlight the large protective impact of not only adding chemoprevention to IRS, but also the protective impact IRS+ITNs have over ITNs alone in this high transmission setting.

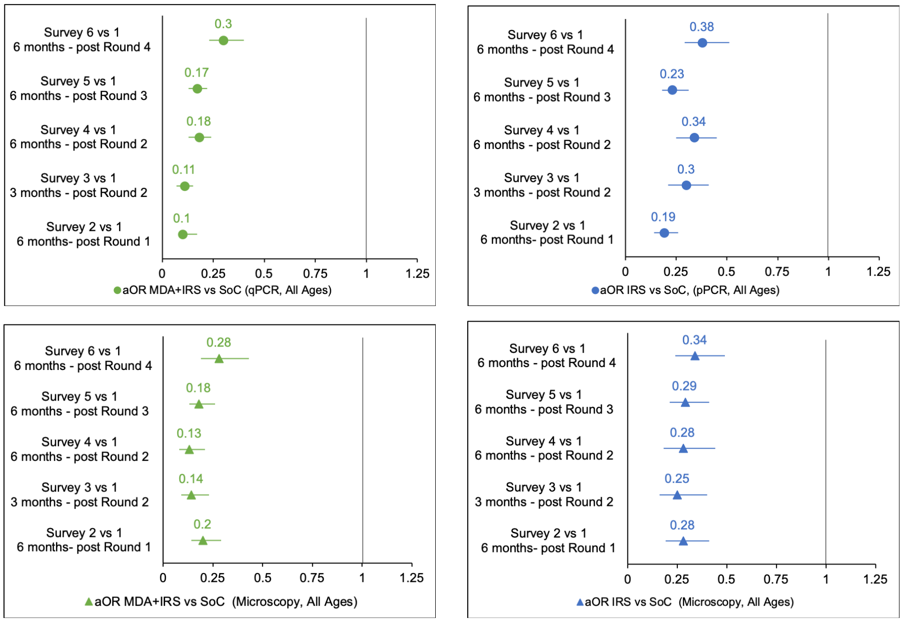

Figure 2 Top left: the DiD adjusted odds ratio for qPCR confirmed malaria in residents of all ages receiving MDA+IRS versus those with standard of care (SoC). Top right: the same DiD adjusted odds ratio for qPCR confirmed malaria in residents of all ages receiving IRS, without MDA, versus those with standard of care. The lower panels display the same as the panels above except for malaria in residents confirmed by microscopy. Horizontal bars indicate 95% confidence intervals.

Appendix III. Malaria prevalence tables

See included excel spreadsheet.
